# Vector Flows That Compute the Capacity of Discrete Memoryless Channels †

**DOI:** 10.3390/e27040362

**Published:** 2025-03-29

**Authors:** Guglielmo Beretta, Marcello Pelillo

**Affiliations:** 1 DAIS, Università Ca’ Foscari di Venezia, Via Torino 155, 30170 Venezia, Italy; 2 DAUIN, Politecnico di Torino, Corso Castelfidardo 34/d, 10138 Torino, Italy; 3 College of Mathematical Medicine, Zhejiang Normal University, Jinhua 321004, China; 4 European Centre for Living Technology, Ca’ Bottacin, Dorsoduro 3911, Calle Crosera, 30123 Venezia, Italy

**Keywords:** analog computation, capacity, convex optimization, discrete memoryless channel, dynamical systems, mutual information, ODE, optimal input distribution, vector flow

## Abstract

One of the fundamental problems of information theory, since its foundation by C. Shannon, has been the computation of the capacity of a discrete memoryless channel, a quantity expressing the maximum rate at which information can travel through the channel. In this paper, we investigate the properties of a novel approach to computing the capacity, based on a continuous-time dynamical system. Interestingly, the proposed dynamical system can be regarded as a continuous-time version of the classical Blahut–Arimoto algorithm, and we can prove that the former shares with the latter an exponential rate of convergence if certain conditions are met. Moreover, a circuit design is presented to implement the dynamics, hence enabling analog computation to estimate the capacity.

## 1. Introduction

Estimating the capacity of a discrete memoryless channel (DMC) is a well-known problem related to the reliability of point-to-point communication systems, as a consequence of C. Shannon’s noisy-channel coding theorem [1,2,3]. A fundamental algorithm to address this problem is the classical Blahut–Arimoto algorithm (BAA), an iterative algorithm based on an alternating maximization procedure [4]. Named after S. Arimoto [5] and R. Blahut [6], who discovered it independently, the BAA requires only some mild conditions on the zero elements of the transition matrix and, unlike its antecedents, it is also applicable when the input alphabet and the output alphabet of the channel have different cardinalities. Notably, the BAA is still a subject of active research (see, e.g., Refs. [7,8,9,10]).

In contrast to the BAA, this paper describes a continuous-time dynamical system used to compute the capacity of a DMC. This dynamical system is obtained via the (forward) flow of a suitable ODE, and it can evolve a given distribution towards an optimal input distribution of the channel, hence enabling capacity computation. In studying this unconventional way to address capacity computation, we were inspired by the work by R. W. Brockett, who, as reported, e.g., in [11], provides a novel way, grounded in calculus, to approach problems traditionally addressed via algorithms [12]. Interestingly, the proposed capacity-computing ODE (denoted by CC-ODE in the sequel) has a connection with the BAA. Indeed, it can be regarded, in a sense, as a continuous-time version of the BAA. To support this claim, we leverage the notion of the multiplicative-weight-update (MWU) rule [13] and its connection to both the BAA (see Refs. [8,14]) and to some discretization techniques used, e.g., in evolutionary game theory [15,16].

The link between the BAA and the CC-ODE flow can be further extended when studying the convergence rates. By construction, the BAA generates a sequence of input distributions, and understanding the convergence rate of this sequence to an optimal input distribution has been central in the study of the BAA ever since its origin [5]. Remarkably, estimating an optimal input distribution for a generic DMC involves some issues affecting not only the BAA, but also any iterative algorithm running on a Turing machine, as recently shown in [10] via computability theory arguments. Despite this, some technical conditions on DMCs ensure exponential convergence of this sequence to an optimal input distribution [5,9]. We prove that under these conditions, in the formulation given in [9], the CC-ODE flow converges exponentially to an optimal input distribution, which can be shown thanks to some tools of Lyapunov’s stability theory [17]. The convergence rate can be further refined for a trivial family of DMCs, namely, the noiseless symmetric channels—see, e.g., Ref. [18] (p. 77). Even though a known formula exists for their capacity, we found it interesting that these channels are associated with a CC-ODE for which an explicit analytic solution is available, and we leverage this to produce a more precise asymptotic estimate of the flow.

Lastly, we propose a circuit design to implement the CC-ODE flow, thereby enabling analog computation of the capacity. Analog computation is an important alternative to digital computation [19], and is still a topic of active research—see, e.g., Refs. [20,21,22,23,24,25,26,27]. We speculate about how the proposed circuit could preserve its effectiveness even in the presence of noise, and we comment on its usage in association with the unavailability of some channel input symbols. See also Ref. [28] for a similar circuit design dealing with a labeling task.

This paper is a follow-up of [29], where the empirical usage of numerical methods applied to the CC-ODE was studied to compute the capacity. However, no mathematical proofs were given in [29], and except for a simplified version of Theorem 2, the results presented in this paper do not appear in [29]. To the best of our knowledge, no previous work has studied continuous-time dynamical systems to compute the capacity of DMCs.

Despite this, the CC-ODE can be regarded, under some technical conditions, as an instance of a class of ODEs discussed in [30], where smooth functions are optimized over polyhedra using some notions of Riemannian geometry. In addition to that, similar ODEs appear in the literature on convex optimization (see, e.g., Ref. [31]), as well as in some models pertaining to evolutionary game theory [16,32]. However, in contrast to [16,30,31], the objective function associated with the CC-ODE may be not differentiable on part of the boundary of the feasible set, and we provide the technical arguments that are required to adapt the existing results to the problem under discussion.

The subsequent sections of this paper are organized as follows. Notation conventions are established in Section 2, and Section 3 reviews the aforementioned class of ODEs that have been used in the literature to tackle optimization programs on a standard simplex. We also mention how relaxed hypotheses on the objective function may negatively affect trajectory convergence to stationary points, and in Lemma 1, we discuss some alternative conditions to overcome this issue. In Section 4, the reader is introduced to the problem of computing the channel capacity for DMCs and its formulation as a concave optimization program on a standard simplex. The main contributions of this paper are in Section 5, which deals with the CC-ODE, its properties, and its link with the BAA, and Section 6, where the convergence rates are discussed. The circuit designed to implement the CC-ODE flow is examined in Section 7. Some simulations are described in Section 8 so as to give more insight into how the CC-ODE flow behaves in comparison with the BAA. Section 9 contains some remarks on our results, and final considerations and future research directions are reported in Section 10.

## 2. Notations

In this paper, the information content is measured in *nats*, since this choice simplifies the theoretical computations involving mutual information, as mentioned, e.g., in [6]. For a conversion to bits, we recall that 1 nat equals ln2 bits [2]. In the sequel, we consider the expression αlnα as well defined and equal to 0 in case α=0. For every integer n>0, the *standard simplex* in $R^{n}$ is the set

$$\Delta_{n}=\left\{\;z\in R^{n}\;|\;z\geq 0\;\mathrm{and}\;\sum_{i=1}^{n}z_{i}=1\;\right\}\;,$$

its (relative) *interior* is the set

$$\mathrm{int}\left(\Delta_{n}\right)=\left\{\;z\in R^{n}\;|\;z>0\;\mathrm{and}\;\sum_{i=1}^{n}z_{i}=1\;\right\}\;,$$

and its (relative) *boundary* is the set $\partial \Delta_{n}=\Delta_{n}\backslash \mathrm{int}\left(\Delta_{n}\right)$. For $z={\left(z_{1},\ldots ,z_{n}\right)}^{⊤}\in R^{n}$, the *support* of x is the set $\mathrm{supp}(z)=\left\{\;i\in [n]|z_{i}\neq 0\;\right\}$. The canonical basis of $R^{n}$ is denoted by $e_{1}$, …$e_{n}$, and $1_{n}=\sum_{i=1}^{n}e_{i}$. Given x, $y\in R^{n}$, we write $\langle x,y\rangle =\sum_{i=1}^{n}x_{i}y_{i}$ for the usual dot product between x and y, whereas we write $∥x∥=\sqrt{\langle x,x\rangle }=\sqrt{\sum_{i=1}^{n}x_{i}^{2}}$ for the Euclidean norm of x, so that ∥x−y∥ denotes the Euclidean distance between x and y. In this paper, gradients are column vectors. Given $x_{1}$, $x_{2}$, …, and $x_{n}\in R$, we define

$$\mathrm{MSQ}\left(x_{1},\ldots x_{n}\right)=\frac{1}{n}\sum_{i=1}^{n}{\left(x_{i}-n^{-1}\sum_{k=1}^{n}x_{k}\right)}^{\;2}\;.$$

## 3. Optimizing Continuous-Time Dynamics

### 3.1. Preliminaries

We recall that a function $v:\Omega \subset R^{n}\to R^{m}$ is *(globally) Lipschitz continuous* on Ω if there exists a constant L>0 such that $∥v\left(x_{1}\right)-v\left(x_{2}\right)∥\leq L∥x_{1}-x_{2}∥$ for every $x_{1}$, $x_{2}\in \Omega$, whereas v is *locally Lipschitz continuous* on Ω if for every x∈Ω, there exists an open neighborhood $O_{x}\subseteq R^{n}$ of x such that the restriction of v to $\Omega \cap O_{x}$ is Lipschitz continuous on $\Omega \cap O_{x}$. Global and local Lipschitz continuity play a fundamental role in the existence and uniqueness results concerning solutions of an ODE, as in Picard–Lindelöf theorem—see, e.g., Refs. [33,34]. Given an open $\Omega \subseteq R^{n}$, a function $v:\Omega \to R^{n}$ that is locally Lipschitz continuous on Ω, consider the ODE

(1) $$\dot{z}=v\left(z\right).$$

We will say that a non-empty set S⊆Ω is *invariant* under (1) if for every y∈S, there exists $z:]-\infty ,+\infty [\to S$ that solves (1) and satisfies z(0)=y. Similarly, we will say that *S* is *forward invariant* under (1) if for every y∈S, there exists $z:[0,+\infty [\to S$ that solves (1) and satisfies z(0)=y. By the assumptions made on v, note that for every y∈S there exists *at most one* solution $z:[0,+\infty [\to \Omega$ of (1) such that z(0)=y. This is a trivial consequence of Picard–Lindelöf theorem.However, note that these assumptions *do not guarantee the existence* of such a solution—which is related to the problem of extending local solutions (see also Ref. [35] (Ch. 17.4))—and we remark that stronger assumptions are generally required to prove the existence, such as those reported in Appendix A. We also recall that a point y∈Ω is *stationary* for (1) if v(y)=0, i.e., if the constant function z(t)≡y solves (1).

### 3.2. Optimizing Differential Equation on the Standard Simplex

A program of the form

(2) $$\max_{z\in \Delta_{n}}\;f\left(z\right)$$

is related, under suitable conditions on its objective function *f*, to the ODE

$$\begin{array}{ccc} {\dot{z}}_{i} & =z_{i}\left[\partial_{i}f\left(z\right)-\sum_{k=1}^{n}z_{k}\partial_{k}f\left(z\right)\right], & \;i\in [n], \end{array}$$

which can also be written in vector notation as

(3) $$\dot{z}=\left[\mathrm{diag}\left(z\right)-zz^{⊤}\right]\nabla f\left(z\right).$$

In particular, for $z\in \mathrm{int}\left(\Delta_{n}\right)$, the ODE (3) is known as the *Shahshahani gradient system* associated with *f*—see, e.g., Refs. [16,32]. Many properties of (3) are discussed in the literature under the assumption that *f* admits a *globally* Lipschitz continuous gradient on $\Delta_{n}$, which guarantees the invariance of $\Delta_{n}$ under (3) and that the function *f* increases strictly along any non-constant solution of (3) evolving on $\Delta_{n}$; see, e.g., Refs. [16,30,31,32,36], and also Appendix B. Indeed, strictly speaking, the condition that is often assumed is that the gradient of *f* is locally Lipschitz continuous on some open superset of $\Delta_{n}$ (see, e.g., Ref. [15]), which is a stronger assumption, as shown, e.g., in [35] (p. 400).

However, as we shall see in the following sections, we are interested in objective functions that can exhibit singularities on $\partial \Delta_{n}$ and so we cannot rely on the usual assumptions made in the literature. Crucially, if the function *f* is differentiable in $\mathrm{int}\left(\Delta_{n}\right)$ but not on some points of $\partial \Delta_{n}$, then forward trajectories of (3) may converge to one of these points, where (3) is undefined, and this may happen even in case additional hypotheses such as concavity are met, as in the example shown in Appendix C. By contrast, this cannot happen under the circumstances described in the next lemma, which we will apply in the sequel:

**Lemma** **1.** *Let $f:R^{n}\to R$ admit a locally Lipschitz continuous gradient on some open $\Omega \subseteq R^{n}$. Suppose f is concave on some non-empty compact convex $K\subseteq \Omega \cap \Delta_{n}$. Let $z:[0,+\infty [\to K$ be a solution of *(3)*.*

(i) There exists $z^{*}=\lim_{t\to +\infty }z\left(t\right)\in K$ and $z^{*}$ is a stationary point for *(3)*;
(ii) If $z\left(0\right)\in \mathrm{int}\left(\Delta_{n}\right)$, then $z^{*}$ is a KKT point for *(2)*;
(iii) If $z\left(0\right)\in \mathrm{int}\left(\Delta_{n}\right)$ and f is concave and continuous on $\Delta_{n}$, then $z^{*}$ is a global solution of *(2)*.

**Proof.**  (i): We now make the following claim, whose technical proof is deferred to Appendix D:

**Claim** **1** (Convergence)**.** There exists $z^{*}=\lim_{t\to +\infty }z\left(t\right)\in K$.

Note that $z^{*}\in K$ entails $z^{*}\in \Omega$; hence, ∇f is well defined and continuous in a neighborhood of $z^{*}$. A standard argument can now be applied to show that $z^{*}$ is stationary for (3)—namely, since the ω-limit here does not escape Ω, then the singleton $\left\{\;z^{*}\;\right\}$ is forward invariant under (3) as a consequence of Gronwall’s lemma—see, e.g., Ref. [16].

(ii): We will use the following claim, which provides a known alternative description, for maximization programs over the standard simplex, of the KKT conditions:

**Claim** **2** (KKT points)**.** A point $x^{*}\in \Delta_{n}$ in which f is differentiable is a KKT point for *(2)* if and only if there exists some λ∈R such that

$\partial_{i}f\left(x^{*}\right)\leq \lambda$ for every i∈[n], with equality for every $i\in \mathrm{supp}(x^{*})$.

The interested reader may find a proof for Claim 2 in Appendix E. Assume $z\left(0\right)\in \mathrm{int}\left(\Delta_{n}\right)$, which entails z(t)>0 for every t>0 by uniqueness of local solutions—see, e.g., Proposition A1.(iii). By (i), the point $z^{*}=\lim_{t\to +\infty }z\left(t\right)$ exists in Ω. Define $\mu \left(z\right)=\sum_{k=1}^{n}z_{k}\partial_{k}f\left(z\right)$ and set $\lambda =\mu (z^{*})$. Since $z^{*}$ is a stationary point for (3), it follows that $\partial_{i}f\left(z^{*}\right)=\lambda$ for every $i\in \mathrm{supp}(z^{*})$. The proof is completed by *reductio ad absurdum* as in the proof of [15] (Prop. 3.5). In fact, let $i\notin \mathrm{supp}(z^{*})$—i.e., let $z_{i}^{*}=0$—and suppose also that $\partial_{i}f\left(z^{*}\right)>\lambda =\mu \left(z^{*}\right)$. Then, by continuity, also $\partial_{i}f\left(y\right)>\mu \left(y\right)$ for every y∈Ω sufficiently close to $z^{*}$; hence, ${\dot{z}}_{i}\left(t\right)>0$ for every t>0 sufficiently large, which contradicts $z_{i}\left(t\right)\to 0^{+}$. It follows that $z^{*}$ is a KKT point for (2) by Claim 2.

(iii): If *f* is concave on $\Delta_{n}$, then every KKT point for (2) is a global solution for (2)—see, e.g., Ref. [37]; hence, (iii) follows by (ii). □

## 4. Problem Formulation

### 4.1. Discrete Memoryless Channel and Capacity

A discrete memoryless channel (DMC) is a communication system that can be described by a triplet C=(X,Y,P), in which $X=\{x_{1},x_{2},\ldots ,x_{n}\}$ and $Y=\{y_{1},y_{2},\ldots ,y_{m}\}$ are finite alphabets called the *input alphabet* and *output alphabet*, respectively, whereas $P={\left[p\left(j|i\right)\right]}_{i\in [n],j\in [m]}\in R^{n\times m}$ is a stochastic matrix, called the *transition matrix*, where p(j|i) expresses the probability that the symbol $y_{j}\in Y$ is observed as output of the system whenever the symbol $x_{i}\in X$ is sent to the system as input [3]. Without loss of generality, we will work under the following assumption:

For every j∈[m], there exists at least one i∈[n] such that p(j|i)>0.

In other words, we are assuming that Y represents the minimal output alphabet required for a description of the DMC. In fact, this assumption ensures that for any selected symbol y∈Y there exists a corresponding input distribution for which *y* occurs as output with positive probability.

Let *X* be the *input variable* of C, i.e., the random variable with range in X modeling the input of the channel, and let *Y* be the *output variable* of C, i.e., the random variable modeling the output of the channel. Following [2], the *mutual information* between *X* and *Y*, denoted by I[X;Y], is a non-negative quantity measuring the reduction in uncertainty about *X* that results from learning the value of *Y*, and its formulation involves the notion of *entropy*, which for a generic discrete random variable *D* with range in a set D is given by $H\left[D\right]=-\sum_{d\in D}p_{D}\left(d\right)\ln\left[p_{D}\left(d\right)\right]$. The random variable *Y* and all the random variables Y|X=x obtained as x∈X appear, together with the *input distribution* $p_{X}$, in the following expression for I[X;Y] (see, e.g., Ref. [2]):

$$I\left[X;Y\right]=H\left[Y\right]-\sum_{x\in X}p_{X}\left(x\right)H\left[Y|X=x\right].$$

The *capacity* of C is the maximum value *C* of I[X;Y] over all possible choices for $p_{X}$:

$$C=\max_{p_{X}}I\left[X;Y\right].$$

The capacity *C* provides a theoretical bound for the information content that can be transmitted through the channel [1,2,3].

### 4.2. Optimization Program for the Capacity

Consider a channel C=(X,Y,P) and set n=|X| and m=|Y|. The maximization problem associated with the capacity admits a well-known formulation as a constrained optimization program over a standard simplex—see, e.g., Ref. [10]. We now show how to derive this program and, in so doing, we take the chance to define some auxiliary functions and parameters that we will extensively use in this paper.

Define the function $q={\left(q_{1},\ldots ,q_{m}\right)}^{⊤}:R^{n}\to R^{m}$, where $q_{j}$ is the linear function

$$z={\left(z_{1},\ldots ,z_{n}\right)}^{⊤}\;\mapsto \;q_{j}\left(z\right)=\sum_{i=1}^{n}p\left(j|i\right)z_{i}.$$

If $z_{i}=p_{X}\left(x_{i}\right)$ for every i∈[n], then $q_{j}\left(z\right)=p_{Y}\left(y_{j}\right)$ for every j∈[m] by the theorem of total probability; thus, z and q=q(z) can be identified with the distributions of *X* and *Y* respectively, and it is possible to write I[X;Y] as a function of z. Indeed, it is sufficient to set

(4) $$\begin{array}{ccc} c_{i} & =\sum_{j=1}^{m}p\left(j|i\right)\ln\left[p\left(j|i\right)\right], & \;i\in [n], \end{array}$$

define the function

(5) $$I\left(z\right)=\sum_{i=1}^{n}c_{i}z_{i}-\sum_{j=1}^{m}q_{j}\ln q_{j},$$

and observe that, for $z\in \Delta_{n}$ corresponding to $p_{X}$, the equality I[X;Y]=I(z) holds. As a result, the capacity *C* of the channel C satisfies

(6) $$C=\max_{z\in \Delta_{n}}\;I\left(z\right)$$

Following [10], we call *optimal input distribution* every global solution to (6), i.e., every $z^{*}\in \Delta_{n}$ such that $I(z^{*})=C$.

## 5. Vector Flow for Capacity Computation

### 5.1. Flow Definition and Its Properties

The function I, which is the objective function of (6), is continuous and concave on $\Delta_{n}$—see, e.g., Ref. [3]. Define the open set

$$\begin{array}{cc} \Omega & =\left\{\;z\in R^{n}|q_{j}\left(z\right)>0\;\mathrm{for}\;\mathrm{all}\;j\in \left[m\right]\;\right\}, \end{array}$$

and note that $\mathrm{int}\left(\Delta_{n}\right)\subset \Omega$.

**Proposition** **1.** *Let z∈Ω. Then,*

(a) *$\partial_{k}I\left(z\right)=c_{k}-1-\sum_{j=1}^{m}p\left(j|k\right)\ln q_{j}\left(z\right)$;*
(b) *$\sum_{k=1}^{n}z_{k}\partial_{k}I\left(z\right)=I\left(z\right)-\sum_{k=1}^{n}z_{k}$;*
(c) *$\partial_{i,k}^{2}I\left(z\right)=-\sum_{j=1}^{m}p\left(j|i\right)p\left(j|k\right){\left[q_{j}\left(z\right)\right]}^{-1}$.*

**Proof.**  See Appendix F. □

In particular, ∇I is well defined and continuous on Ω, and so ∇I is locally Lipschitz continuous on Ω. However, note that I is singular on ∂Ω.

Now, consider the ODE

(7) $$\begin{array}{ccc} {\dot{z}}_{i}\; & =z_{i}\left[\partial_{i}I\left(z\right)-\sum_{k=1}^{n}z_{k}\partial_{k}I\left(z\right)\right],\; & \;i\in [n]. \end{array}$$

We will also refer to Equation (7) as the capacity computing ODE (CC-ODE for short).

**Theorem** **1** (Forward invariance)**.** *The set $\Omega \cap \Delta_{n}$ is forward invariant under *(7)*.*

**Proof.**  Let

$$Y_{0}=\left\{\;j\in [m]|p(j|i)=0\;\mathrm{for}\;\mathrm{some}\;i\in [n]\;\right\}.$$

It is easy to see that for every j∈[m] and every $z\in \Delta_{n}$:

- If $j\notin Y_{0}$, then $q_{j}\left(z\right)\geq \min_{i\in [n]}p(j|i)>0$;
- If $j\in Y_{0}$, then $q_{j}\left(z\right)=0$ if and only if $\mathrm{supp}\left(z\right)\cap S_{j}=\emptyset$, where the set $S_{j}\subseteq \left[n\right]$ is defined by $S_{j}=\left\{\;i\in [n]|p(j|i)\neq 0\;\right\}$.

For every $j\in Y_{0}$, we define some the following objects:

- The vector $b^{\left(j\right)}=\sum_{i=1}^{n}p(j|i)e_{i}$;
- The function $\beta_{j}:\Omega \to R$ given by
  (8) $$\beta_{j}\left(z\right)=\langle b^{\left(j\right)},\left[\mathrm{diag}\left(z\right)-zz^{⊤}\right]\nabla I\left(z\right)\rangle ;$$
- The real number $\varepsilon_{j}$ as a positive solution to the system of inequalities
  (9) $$c_{i}-p\left(j|i\right)\ln\varepsilon_{j}-C>0\;\mathrm{for}\;\mathrm{all}\;i\in S_{j},$$
  where $c_{i}$ and *C* are defined as in (4), (6) (note that such an $\varepsilon_{j}$ exists, since −lnu→+∞ as $u\to 0^{+}$ and p(j|i)≠0 for $i\in S_{j}$).

We make the following claim:

**Claim** **3.** *Let $z\in \Omega \cap \Delta_{n}$ and let $j\in Y_{0}$. Then,*

(10) $$0<q_{j}\left(z\right)\leq \varepsilon_{j}\Rightarrow \beta_{j}\left(z\right)\geq 0.$$

**Proof** **of Claim 3.** By Proposition 1, the definition of $S_{j}$, and using that $\sum_{i=1}^{n}z_{i}=1$,

$$\begin{array}{cc} \beta_{j}\left(z\right)\; & =\sum_{i=1}^{n}p\left(j|i\right)z_{i}\left[\partial_{i}I\left(z\right)-\sum_{k=1}^{n}z_{k}\partial_{k}I\left(z\right)\right]\\ \; & =\sum_{i=1}^{n}p\left(j|i\right)z_{i}\left\{c_{i}-\sum_{J=1}^{m}p\left(J|i\right)\ln\left[q_{J}\left(z\right)\right]-I\left(z\right)\right\}\\ \; & =\sum_{i\in S_{j}}p\left(j|i\right)z_{i}\left\{c_{i}-\sum_{J=1}^{m}p\left(J|i\right)\ln\left[q_{J}\left(z\right)\right]-I\left(z\right)\right\}, \end{array}$$

and so

(11) $$\beta_{j}\left(z\right)\geq \sum_{i\in S_{j}}p\left(j|i\right)z_{i}\left\{c_{i}-p\left(j|i\right)\ln\left[q_{j}\left(z\right)\right]-C\right\},$$

since $z\in \Delta_{n}$ entails both I(z)≤C and $\ln\left[q_{J}\left(z\right)\right]\leq 0$ for every *J*. If $q_{j}\left(z\right)\leq \varepsilon_{j}$, then $\ln q_{j}\left(z\right)\leq \ln\varepsilon_{j}$, and so, by (9) and (11), it follows that $\beta_{j}\left(z\right)\geq 0$. □

Now, let $y\in \Omega \cap \Delta_{n}$. We have to prove that a solution $z:[0,+\infty [\to \Omega \cap \Delta_{n}$ of (7) exists satisfying z(0)=y. We first make the following claim:

**Claim** **4.**  *There exists a convex compact $K\subseteq \Omega \cap \Delta_{n}$ such that y∈K, and K is forward invariant under *(7)*.*

**Proof** **of Claim 4.** For every $j\in Y_{0}$, set $\alpha_{j}=\min\left\{\;\varepsilon_{j},q_{j}\left(y\right)\;\right\}$ and define

$$K=\left\{\;z\in \Delta_{n}|q_{j}\left(z\right)\geq \alpha_{j}\;\mathrm{for}\;\mathrm{every}\;j\in Y_{0}\;\right\}.$$

By construction, $y\in K\subseteq \Omega \cap \Delta_{n}$. Note that *K* is a compact and convex polytope containing the elements $z\in R^{n}$ that satisfy the following constraints:

- $\langle e_{i},z\rangle \geq 0$ for every i∈[n];
- $\langle 1_{n},z\rangle =1$;
- $\langle b^{\left(j\right)},z\rangle \geq \alpha_{j}$ for every $j\in Y_{0}$.

Therefore, for every z∈K, the *tangent cone* TK(z)—see Appendix A or, e.g., Ref. [16]—is the set of $u\in R^{n}$ such that

(a) $u_{i}\geq 0$ for every i∉supp(z);
(b) $\sum_{i=1}^{n}u_{i}=0$;
(c) For every $j\in Y_{0}$, if $\langle b^{\left(j\right)},z\rangle =\alpha_{j}$ then $\langle b^{\left(j\right)},u\rangle \geq 0$.

Note now that $\left[\mathrm{diag}\left(z\right)-zz^{⊤}\right]\nabla I\left(z\right)\in TK\left(z\right)$ for every z∈K. In fact, (a) and (b) are immediate to check, whereas (c) holds by Claim 3. The thesis follows by Theorem A1 in Appendix A. □

By Claim 4, there exists a compact $K\subseteq \Omega \cap \Delta_{n}$ and a solution $z:[0,+\infty [\to K\subseteq \Omega \cap \Delta_{n}$ of (7) satisfying z(0)=y. □

By Theorem 1, we can define a continuous-time dynamical system on $\Omega \cap \Delta_{n}$ via the (forward) *flow* generated on $\Omega \cap \Delta_{n}$ by the CC-ODE, i.e., via the function

(12) $$\varphi_{I}:\Omega \cap \Delta_{n}\times [0,+\infty [\to \Omega \cap \Delta_{n}$$

such that for every $y\in \Omega \cap \Delta_{n}$, the function $z=\varphi_{I}\left(y,\cdot \right)$ is the unique solution of

(13) $$\left\{\begin{array}{cc} \dot{z} & =\left[\mathrm{diag}\left(z\right)-zz^{⊤}\right]\nabla I\left(z\right),\;t\geq 0\\ z(0) & =y. \end{array}\right.$$

**Proposition** **2** (CC-ODE flow properties)**.** *Let $y\in \Omega \cap \Delta_{n}$ and set $z=\varphi_{I}\left(y,\cdot \right)$.*

(a) *The equality supp(z(t))=supp(y) holds for every t≥0;*
(b) *Either y is a stationary point for *(7)* or I∘z is strictly increasing;*
(c) There exists $z^{*}=\lim_{t\to +\infty }z\left(t\right)\in \Omega \cap \Delta_{n}$, and $z^{*}$ is a stationary point for *(7)*;
(d) The restriction of I to the set $\Gamma =\left\{\;x\in \Delta_{n}|\mathrm{supp}(x)\subseteq \mathrm{supp}(y)\;\right\}$ attains its maximum in $z^{*}$:
  (14) $$z^{*}\in \arg\max_{z\in \Gamma }I(z);$$

**Proof.**  (a), (b): These properties hold for Shahshahani gradient systems trajectories, and similar proofs are valid in our setting by the uniqueness of local solutions of (7). For more details, see Appendix B.

(c): This follows from Claim 4 and Lemma 1.(i).

(d): Assume without loss of generality that only the first $\tilde{n}$ coordinates of y are positive, where $0<\tilde{n}\leq n$. Consider the injection $\iota :R^{\tilde{n}}\to R^{n}$ given by $\iota {\left(w\right)}^{⊤}=\left(w^{⊤},0^{⊤}\right)$, and define the function $I^{\prime }=I\circ \iota$. Observe that z=ι∘x by uniqueness of solutions, where $x:[0,+\infty [\to \Delta_{\tilde{n}}$ solves

$$\left\{\begin{array}{cc} \dot{x} & =\left[\mathrm{diag}\left(x\right)-xx^{⊤}\right]\nabla I^{\prime }\left(x\right),\;t\geq 0\\ x(0) & =\iota^{-1}\left(y\right). \end{array}\right.$$

Since $\Gamma =\iota (\Delta_{\tilde{n}})$ and $\iota^{-1}\left(y\right)\in \mathrm{int}\left(\Delta_{\tilde{n}}\right)$ by construction, the result follows by Lemma 1 applied to $f=I^{\prime }$. □

In particular, Proposition 2 gives the following fundamental theorem:

**Theorem** **2** (CC-ODE flow-attaining capacity)**.** *Let $y\in \mathrm{int}\left(\Delta_{n}\right)$. Then $\lim_{t\to +\infty }\varphi_{I}\left(y,t\right)$ exists and is an optimal input distribution. Either $\varphi_{I}\left(y,\cdot \right)\equiv y$ is a constant function, or $I\left(\varphi_{I}\left(y,t\right)\right)$ is strictly increasing in t and converges to C as t→+∞.*

**Proof.**  The proof follows directly from Proposition 2. □

### 5.2. Connection with Blahut–Arimoto Algorithm

The BAA is an iterative algorithm that can be described via a map $\Phi_{\mathrm{BA}}:\Delta_{n}\to \Delta_{n}$, here called the *Blahut–Arimoto map*. Given an initial input distribution $z^{\left(0\right)}\in \mathrm{int}\left(\Delta_{n}\right)$, Blahut [6] and Arimoto [5] proved that the sequence ${\left\{z^{\left(k\right)}\right\}}_{k}$ defined by $z^{\left(k+1\right)}=\Phi_{\mathrm{BA}}\left(z^{\left(k\right)}\right)$ satisfies $I(z^{\left(k\right)})\to C$ as k→∞. It has been observed [8] that $\Phi_{\mathrm{BA}}$ acts according to the MWU rule  [13]:

(15) $$z_{i}^{\left(k+1\right)}=z_{i}^{\left(k\right)}\frac{\exp[w_{i}\left(z^{\left(k\right)}\right)]}{\sum_{\ell =1}^{n}z_{\ell }^{\left(k\right)}\exp\left[w_{\ell }\left(z^{\left(k\right)}\right)\right]},$$

where the weights $w_{1}\left(z\right)$, …, $w_{n}\left(z\right)$ satisfy

(16) $$w_{i}\left(z\right)=\sum_{j\in [m]:p(j|i)\neq 0}p\left(j|i\right)\ln\left(\frac{p(j|i)}{q_{j}\left(z\right)}\right).$$

Interestingly, there exists a numerical scheme used for approximating Shahshahani gradient systems that is also based on MWU, which is the following:

(17) $$z_{i}^{\left(k+1\right)}=z_{i}^{\left(k\right)}\frac{\exp\left[\tau \partial_{i}f\left(z^{\left(k\right)}\right)\right]}{\sum_{\ell =1}^{n}z_{\ell }^{\left(k\right)}\exp\left[\tau \partial_{\ell }f\left(z^{\left(k\right)}\right)\right]},$$

where τ>0 is the stepsize and *f* must be sufficiently smooth. The recurrence described in (17) is well known in evolutionary game theory—see, e.g., the deduction of the discrete replicator-dynamics model [15]. One fundamental property of (17) is that if $z^{\left(k\right)}$ is an element of $\Delta_{n}$ and the gradient of *f* is defined in $z^{\left(k\right)}$, then (17) defines $z^{\left(k+1\right)}$ as an element of $\Delta_{n}$ having the same support of $z^{\left(k\right)}$, in agreement with the support invariance of the continuous-time dynamics—see also Appendix B. In particular, if $z^{\left(0\right)}\in \mathrm{int}\left(\Delta_{n}\right)$ and *f* is differentiable in $\mathrm{int}\left(\Delta_{n}\right)$, then the recurrence given by (17) can be used to produce a sequence ${\left\{z^{\left(k\right)}\right\}}_{k}$ with arbitrary length (even though, in numerical implementations, the recurrence initialized in $\mathrm{int}\left(\Delta_{n}\right)$ could generate points on $\partial \Delta_{n}$ due to floating-point arithmetic, see also Ref. [29]).

We can now explain in the following theorem how the CC-ODE flow can be regarded as a continuous-time version of the BAA:

**Theorem** **3.** *The Blahut-Arimoto map coincides with the MWU rule *(17)* applied to f=I defined as in *(5)* with stepsize τ=1.*

**Proof.**  Observe that

(18) $$\partial_{i}I\left(z\right)=-1+w_{i}\left(z\right),$$

with $w_{i}\left(z\right)$ defined as in (16); hence, $z^{\left(k+1\right)}=\Phi_{\mathrm{BA}}\left(z^{\left(k\right)}\right)$ satisfies the following:

$$\begin{array}{cc} z_{i}^{\left(k+1\right)}\; & =z_{i}^{\left(k\right)}\frac{\exp\left[\partial_{i}I\left(z^{\left(k\right)}\right)+1\right]}{\sum_{\ell =1}^{n}z_{\ell }^{\left(k\right)}\exp\left[\partial_{\ell }I\left(z^{\left(k\right)}\right)+1\right]}\\ \; & =z_{i}^{\left(k\right)}\frac{\exp\left[\partial_{i}I\left(z^{\left(k\right)}\right)\right]}{\sum_{\ell =1}^{n}z_{\ell }^{\left(k\right)}\exp\left[\partial_{\ell }I\left(z^{\left(k\right)}\right)\right]}. \end{array}$$

□

## 6. Convergence Rate

### 6.1. Conditions for Exponential Convergence

Consider an optimal input distribution $z^{*}$. We now present some conditions that ensure that the flow converges exponentially to $z^{*}$. To this end, we will study a first-order approximation of

(19) $$v\left(z\right)=\left[\mathrm{diag}\left(z\right)-zz^{⊤}\right]\nabla I\left(z\right)$$

in the vicinity of $z^{*}$. In our continuous-time scenario, this corresponds to what is examined in [9], where a truncated Taylor expansion of $\Phi_{\mathrm{BA}}\left(z\right)$ in $z^{*}$ is studied to investigate the rate of convergence of the sequence given by $z^{\left(k+1\right)}=\Phi_{\mathrm{BA}}\left(z^{\left(k\right)}\right)$ to $z^{*}$, where $z^{\left(0\right)}\in \mathrm{int}\left(\Delta_{n}\right)$. To this end, we consider a classification over [n] introduced in [9] that involves the coordinates of $z^{*}$ and $\nabla I(z^{*})$. Specifically, an index i∈[n] is classified as follows (note that the case $\partial_{i}I\left(z^{*}\right)>C-1$ is excluded by the KKT conditions):

- *type* I if $i\in \mathrm{supp}(z^{*})$ and $\partial_{i}I\left(z^{*}\right)=C-1$;
- *type* II if $i\notin \mathrm{supp}(z^{*})$ and $\partial_{i}I\left(z^{*}\right)=C-1$;
- *type* III if $i\notin \mathrm{supp}(z^{*})$ and $\partial_{i}I\left(z^{*}\right)<C-1$.

In particular, we remark that the vector $\mu \in R^{n}$ given by

(20) $$\mu ={\left(\mu_{1},\ldots ,\mu_{n}\right)}^{T}=\sum_{i=1}^{n}\left[C-1-\partial_{i}I\left(z^{*}\right)\right]\;e_{i}$$

satisfies $\mu_{i}>0$ if *i* is of type III, and $\mu_{i}=0$ otherwise. As shown in the following proposition, this classification helps in the study of the first-order approximation of (19) near $z^{*}$:

**Proposition** **3.** *Let i∈[n]. Then,*

$$\nabla v_{i}\left(z^{*}\right)=\left\{\begin{array}{cc} z_{i}^{*}\left[\nabla \partial_{i}I\left(z^{*}\right)-\nabla I\left(z^{*}\right)+1_{n}\right] & \mathrm{if}\;i\;\mathrm{is}\;\mathrm{of}\;\mathrm{type}\;I,\\ 0 & \mathrm{if}\;i\;\mathrm{is}\;\mathrm{of}\;\mathrm{type}\;\mathrm{II},\\ \partial_{i}I\left(z\right)-I\left(z\right)+1]e_{i} & \mathrm{if}\;i\;\mathrm{is}\;\mathrm{of}\;\mathrm{type}\;\mathrm{III}. \end{array}\right.$$

**Proof.**  By Proposition 1,

$$\begin{array}{cc} \nabla v_{i}\left(z\right)= & \nabla \left\{z_{i}\left[\partial_{i}I\left(z\right)-I\left(z\right)+\sum_{k=1}^{n}z_{k}\right]\right\}\\ = & \left[\partial_{i}I\left(z\right)-I\left(z\right)+\sum_{k=1}^{n}z_{k}\right]e_{i}+z_{i}\left[\nabla \partial_{i}I\left(z\right)-\nabla I\left(z\right)+1_{n}\right], \end{array}$$

and note that $I(z^{*})=C$. □

By Proposition 3, if *i* is of type II, then $v_{i}$ has a null gradient, and higher-order terms would be required to study $v_{i}$ in the vicinity of $z^{*}$. Indeed, in order to obtain a non-singular linearization of (7) in a neighborhood of $z^{*}$, we will make the following assumption:

(N1) For some positive $n^{\prime }\leq n$, the indices i=1, …, $n^{\prime }$ are of type I and the remaining $n^{\prime \prime \prime }=n-n^{\prime }$ indices are of type III.

Similarly to what is done in [9], it is useful to introduce some additional notation to distinguish between indices of different types. To this end, for every $x\in R^{n}$, define $P_{I}x={\left(x_{1},\ldots ,x_{n^{\prime }}\right)}^{⊤}\in R^{n^{\prime }}$ and $P_{\mathrm{III}}x={\left(x_{n^{\prime }+1},\ldots ,x_{n}\right)}^{⊤}\in R^{n^{\prime \prime \prime }}$ so that $x^{⊤}=\left(P_{I}x^{⊤},P_{\mathrm{III}}x^{⊤}\right)$. Note that ${\left(z^{*}\right)}^{⊤}=\left({\left(P_{I}z^{*}\right)}^{⊤},0^{⊤}\right)$ and $\mu^{⊤}=(0^{⊤},{\left(P_{\mathrm{III}}\mu \right)}^{⊤})$. Moreover, let H be the Hessian of I in $z^{*}$, and write the submatrix of H containing its first $n^{\prime }$ rows as $\left[H_{I,I}|H_{I,\mathrm{III}}\right]$, where $H_{I,I}\in R^{n^{\prime }\times n^{\prime }}$ and $H_{I,\mathrm{III}}\in R^{n^{\prime }\times n^{\prime \prime \prime }}$.

**Theorem** **4.** Assume *(N1)*. Define $Z=\mathrm{diag}\left(P_{I}z^{*}\right)\in R^{n^{\prime }\times n^{\prime }}$, let $D=\mathrm{diag}\left(P_{\mathrm{III}}\mu \right)\in R^{n^{\prime \prime \prime }\times n^{\prime \prime \prime }}$, and let $J\in R^{n\times n}$ be the Jacobian matrix of v in $z^{*}$. Then $J\left(z-z^{*}\right)=M\left(z-z^{*}\right)$ for every $z\in \Delta_{n}$, where $M\in R^{n\times n}$ is the upper-triangular block matrix

(21) $$M=\left[\begin{array}{cc} ZH_{I,I} & ZH_{I,\mathrm{III}}+\left(P_{I}z^{*}\right){\left(P_{\mathrm{III}}\mu \right)}^{⊤}\\ \;ine\; & -D \end{array}\right].$$

**Proof.**  For every i∈[n], the *i*-th row of H is precisely $\nabla v_{i}{\left(z\right)}^{⊤}$. Apply Proposition 3, using the fact that $\sum_{i=1}^{n}z_{i}-z_{i}^{*}=1-1=0$. □

Before proving our main result on the convergence rate of the CC-ODE flow, we need the following additional assumption:

(N2) The first $n^{\prime }$ rows of the transition matrix P are independent.

**Theorem** **5** (Exponential convergence rate)**.** Suppose *(N1)* and *(N2)* hold. Then the maximum eigenvalue $\lambda_{\max}$ of M is negative. Moreover, $z^{*}$ is the only optimal input distribution for the channel C, and for every $0<\alpha <\;|\lambda_{\max}|$, there exists δ, K>0 such that for every $y\in \Omega \cap \Delta_{n}$, if $∥y-z^{*}∥<\delta$, then

(22) $$∥\varphi_{I}\left(y,t\right)-z^{*}∥\leq K∥y-z^{*}∥\exp\left(-\alpha t\right)$$

*for every t≥0.*

**Proof.**  Consider first the matrix M defined in (21), whose eigenvalues are the union, counting multiplicity, of the eigenvalues of $ZH_{I,I}$ and those of −D. The eigenvalues of −D are $-\mu_{n^{\prime }+1}$, …, $-\mu_{n}$, whereas those of $ZH_{I,I}$ are described in the following lemma, proved in [9] (Section III.C):

**Lemma** **2** (Nakagawa et al. [9])**.** *The matrix $ZH_{I,I}$ is diagonalizable, has eigenvalues $-1=\lambda_{1}\leq \ldots \leq \lambda_{n^{\prime }}\leq 0$, and $\lambda_{n^{\prime }}<0$ if and only if the first $n^{\prime }$ rows of the transition matrix P are independent.*

By Lemma 2 and Assumption (N2), it follows that $\lambda_{\max}<0$.

The remaining part of the proof relies on a standard application of Lyapunov’s stability theory (see, e.g., Refs. [17,38]), combined with the application of Theorem 4. Given $0<\alpha <\;|\lambda_{\max}|$, the matrix $\tilde{M}=M+\alpha I$ has only real negative eigenvalues by construction. Consequently, the Lyapunov’s equation [17]

(23) $${\tilde{M}}^{⊤}B+B\tilde{M}=-I$$

is solved by a positive definite symmetric matrix $B\in R^{n\times n}$. Consider the quadratic form V(x)=〈x,Bx〉. Calling the minimum and the maximum eigenvalue of B respectively *a* and *A*, it follows that 0<a<A and

(24) $$\begin{array}{cc} a{∥x∥}^{2} & \leq V\left(x\right)\leq A{∥x∥}^{2}. \end{array}$$

Recall that

$$v\left(z\right)=J\left(z-z^{*}\right)+r\left(z-z^{*}\right)$$

where J is the Jacobian matrix of v in $z^{*}$ and

(25) $$\begin{array}{ccccc} \; & \frac{∥r\left(z-z^{*}\right)∥}{∥z-z^{*}∥}\to 0 & \;\mathrm{as}\; & \; & z\to z^{*}. \end{array}$$

Therefore, setting $z\left(t\right)=\varphi_{I}\left(y,t\right)$ and performing the substitution $\eta \left(t\right)=z\left(t\right)-z^{*}$ yields

$$\begin{array}{cc} \frac{d}{dt}V\left(\eta \right) & =2\langle v(z),B\eta \rangle \\ \; & =2\langle \eta ,BJ\eta \rangle +2\langle r(\eta ),B\eta \rangle \\ \; & =2\langle \eta ,BM\eta \rangle +2\langle r(\eta ),B\eta \rangle , \end{array}$$

where the last equality is a consequence of Theorem 4. By definition of $\tilde{M}$, by (23), and using that $B=B^{⊤}$,

$$\begin{array}{cc} 2\langle \eta ,BM\eta \rangle \; & =2\langle \eta ,B\tilde{M}\eta \rangle -2\alpha \langle \eta ,B\eta \rangle \\ \; & =\langle \eta ,\left({\tilde{M}}^{⊤}B+B\tilde{M}\right)\eta \rangle -2\alpha V\left(\eta \right)\\ \; & =-{∥\eta ∥}^{2}-2\alpha V\left(\eta \right). \end{array}$$

Hence,

$$\begin{array}{cc} \frac{d}{dt}V\left(\eta \right)\; & =-{∥\eta ∥}^{2}-2\alpha V\left(\eta \right)+2\langle r\left(\eta \right),B\eta \rangle \\ \; & =-\left(1-2\langle \frac{r(\eta )}{∥\eta ∥},\frac{B\eta }{∥\eta ∥}\rangle \right){∥\eta ∥}^{2}-2\alpha V\left(\eta \right). \end{array}$$

Suppose now that for some T>0, the expression

(26) $$1-2\langle \frac{r(\eta )}{∥\eta ∥},\frac{B\eta }{∥\eta ∥}\rangle$$

is positive for every $t\in [0,T[$. Then, the inequality d/dt[V(η)]≤−2αV(η) holds on $[0,T[$. By Gronwall’s lemma [17], this implies that $V\left(\eta (t)\right)\leq V\left(\eta (0)\right)\exp\left(-2\alpha t\right)$, and so by (24)

(27) $$∥\eta \left(t\right)∥\leq \sqrt{\frac{A}{a}}∥\eta \left(0\right)∥\exp\left(-\alpha t\right)$$

for every $t\in [0,T[$. What is left is proving that for δ>0 sufficiently small, if $∥y-z^{*}∥=∥\eta \left(0\right)∥<\delta$, then (26) is positive for every t≥0. This follows easily from (25) and (27).

Finally, to prove that $z^{*}$ is the unique optimal input distribution, suppose by absurd that there exists an optimal input distribution $y^{*}\neq z^{*}$. Note that, by convexity of I, the set of optimal input distributions is a convex subset of $\Delta_{n}$. Then every convex combination of $y^{*}$ and $z^{*}$ is an optimal input distribution. In particular, infinitely many stationary points exist whose distance from $z^{*}$ does not exceed δ, and this contradicts the definition of δ. □

Theorem 5 is a continuous-time counterpart of the following result reported in [9]:

**Theorem** **6** (Nakagawa et al. [9])**.** Suppose *(N1)* and *(N2)* hold, and that there exists a unique optimal input distribution $z^{*}$ for the channel C. Define $\vartheta_{\max}$ as the maximum of the set $\left\{\;1+\lambda_{i}|\lambda_{i}\in \sigma \left(ZH\right)\;\right\}\cup \left\{\;e^{-\mu_{i}}|i=n^{\prime }+1,\ldots n\;\right\}$. Then for every $\vartheta_{\max}<\vartheta <1$, there exists δ, K>0 such that for every $y\in \mathrm{int}\left(\Delta_{n}\right)$, if $∥y-z^{*}∥<\delta$, then

(28) $$∥\Phi_{\mathrm{BA}}^{\circ N}\left(y\right)-z^{*}∥\leq K\vartheta^{N}∥y-z^{*}∥.$$

### 6.2. Noiseless Symmetric Channels

In this section, we refine the result given in Theorem 5 under the additional hypothesis that C is a noiseless symmetric channel. Following [18] (pp. 77–78), we recall that the channel C=(X,Y,P) is called

- *Deterministic* if the output *Y* is a deterministic function of the input *X*.
- *Lossless* if the input *X* is completely determined by the output *Y*.
- *Noiseless* if it is both deterministic and lossless.
- *Symmetric* if in the transition matrix P every row is a permutation of every other row, and every column is a permutation of every other column.

We now assume that C is noiseless and symmetric. Then |Y|=|X|=n and, up to a suitable reordering of the output alphabet, we may assume that the transition matrix is the n×n identity matrix. Note that q=z in this case, and so $\Omega ={]0,+\infty [}^{n}$, the objective function is

$$I\left(z\right)=-\sum_{i=1}^{n}z_{i}\ln z_{i},$$

and the CC-ODE is

(29) $$\begin{array}{ccc} \dot{z_{i}} & =-z_{i}\left(\ln z_{i}-I\left(z\right)+1-\sum_{k=1}^{n}z_{k}\right), & \;i\in [n]. \end{array}$$

The channel admits a unique optimal input distribution, which is $b=n^{-1}1_{n}$, the barycenter of $\Delta_{n}$; thus C=I(b)=lnn—see, e.g., Ref. [18] (pp. 84–85). In addition to that, it is easy to verify an interesting property of noiseless symmetric channels, namely that, for these channels, the BAA requires at most one iteration to find the optimal input distribution. By Theorem 2, we deduce that, for $y\in \mathrm{int}\left(\Delta_{n}\right)\backslash \left\{b\right\}$, we have $\varphi_{I}\left(y,t\right)\to b$ as t→+∞, and also $\varphi_{I}\left(b,t\right)\equiv b$. We will see that a more precise asymptotic estimate on $\varphi_{I}$ is available by leveraging the explicit solution of (29) on $\mathrm{int}\left(\Delta_{n}\right)$.

**Theorem** **7.** *If C is the noiseless channel, then, for every $y\in \mathrm{int}\left(\Delta_{n}\right)\backslash \left\{b\right\}$,*

(30) $$∥\varphi_{I}\left(y,t\right)-b∥∼e^{-t}{\left[\frac{\mathrm{MSQ}(\ln y_{1},\ldots \ln y_{n})}{n}\right]}^{\;1/2}$$

*as t→+∞.*

**Proof.**  Given $y\in \mathrm{int}\left(\Delta_{n}\right)$, the function $z\left(t\right)=\varphi_{I}\left(y,t\right)$ is given by the following explicit analytical expression:

(31) $$z_{i}\left(t\right)=\frac{\exp\left(e^{-t}\ln y_{i}\right)}{\sum_{k=1}^{n}\exp\left(e^{-t}\ln y_{k}\right)}.$$

Note that $e^{-t}\to 0$ as t→+∞; hence, using the McLaurin expansion of the exponential function,

$$\begin{array}{cc} z_{i}-n^{-1} & =\frac{n\exp\left(e^{-t}\ln y_{i}\right)-\sum_{k=1}^{n}\exp\left(e^{-t}\ln y_{k}\right)}{n\sum_{k=1}^{n}\exp\left(e^{-t}\ln y_{k}\right)}\\ \; & =\frac{n\left(1+e^{-t}\ln y_{i}\right)-\sum_{k=1}^{n}\left(1+e^{-t}\ln y_{k}\right)+o\left(e^{-t}\right)}{n\left[\sum_{k=1}^{n}1+o\left(1\right)\right]}\\ \; & =\frac{n\ln y_{i}-\sum_{k=1}^{n}\ln y_{k}+o\left(1\right)}{n^{2}+o\left(1\right)}\;e^{-t}. \end{array}$$

Therefore,

$$\begin{array}{c} n^{2}e^{2t}{∥z\left(t\right)-b∥}^{2}=\sum_{i=1}^{n}{\left[ne^{t}\left(z_{i}-n^{-1}\right)\right]}^{2}\\ \;\to \sum_{i=1}^{n}{\left(\ln y_{i}-n^{-1}\sum_{k=1}^{n}\ln y_{k}\right)}^{2}=n\mathrm{MSQ}\left(\ln y_{1},\ldots \ln y_{n}\right), \end{array}$$

which gives (30). □

## 7. Analog Implementation

**Figure 1:**
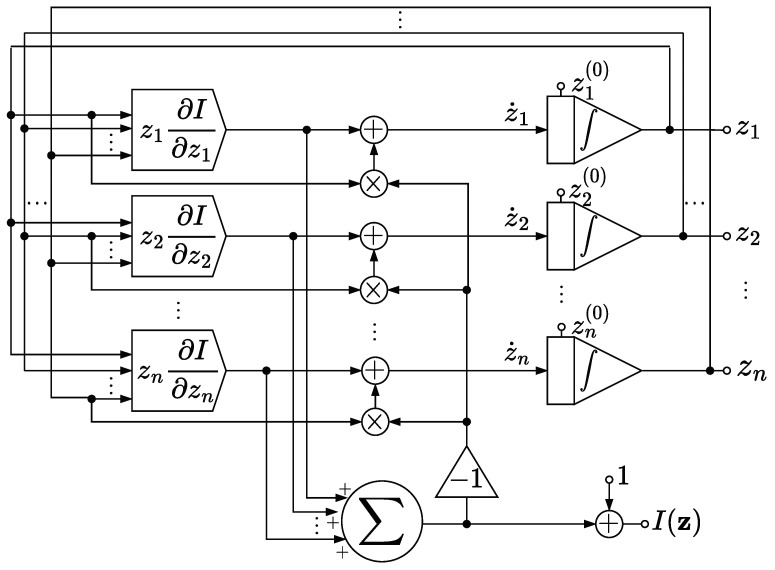
Ideal circuit.

An attractive feature of continuous-time methods is their amenability to be mapped onto hardware circuits [39,40]. Figure 1 depicts a circuit implementing the CC-ODE flow to compute analogically the capacity of a DMC.

The circuit requires $z_{1}\left(0\right)$, …, $z_{n}\left(0\right)$ as input, which represent the coordinates of a point $z\left(0\right)\in \mathrm{int}\left(\Delta_{n}\right)$ in which a trajectory of (7) is initialized. Moreover, the circuit requires, for every k∈[n], a module to implement $z\mapsto z_{k}\partial_{k}I\left(z\right)$, which we here consider as a black-box. These modules encode the dependence of the circuit on the transition matrix P of the DMC. We remark on the following feature of these modules:

**Proposition** **4.** *For every k∈[n], the map $\Omega \cap \Delta_{n}∋z\mapsto z_{k}\partial_{k}I\left(z\right)$ can be extended to a continuous function defined on $\Delta_{n}$.*

**Proof.**  Consider the function L:[0,1]→R given by L(u)=ulnu for 0<u≤1 and L(0)=0, which is a continuous function on [0,1]. Then, by Proposition 1, for every $z\in \Omega \cap \Delta_{n}$,

$$\begin{array}{cc} z_{k}\partial_{k}I\left(z\right) & =z_{k}\left\{c_{k}-1-\sum_{j=1}^{m}p\left(j|k\right)\ln\left[q_{j}\left(z\right)\right]\right\}\\ \; & =z_{k}\left(c_{k}-1\right)-\sum_{j=1}^{m}\frac{z_{k}p\left(j|k\right)}{q_{j}\left(z\right)}L\left(q_{j}\left(z\right)\right), \end{array}$$

and since $q_{j}\left(z\right)=\sum_{i=1}^{n}z_{i}p\left(j|i\right)\geq z_{k}p\left(j|k\right)\geq 0$, it follows that $z\mapsto z_{k}\partial_{k}I\left(z\right)$ is bounded and admits a continuous extension to $\Delta_{n}$, which is given by

$$z\mapsto z_{k}\left\{c_{k}-1-\sum_{j\in \left[m\right]\;:\;q_{j}\left(z\right)\neq 0}p\left(j|k\right)\ln\left[q_{j}\left(z\right)\right]\right\}.$$

 □

The circuit outputs $z={\left(z_{1},\ldots ,z_{n}\right)}^{⊤}$, which at time t=0 coincides with z(0), and thanks to the integrator elements appearing in the circuit [40], the recurrent design of the circuit evolves z according to Volterra’s equation [33]:

$$z_{i}\left(t\right)=z_{i}\left(0\right)+\int_{0}^{t}\;\left[z_{i}\left(s\right)\partial_{i}I\left(z(s)\right)-z_{i}\left(s\right)\sum_{k=1}^{n}z_{k}\left(s\right)\partial_{k}I\left(z(s)\right)\right]ds.$$

Hence, $z\left(t\right)=\varphi_{I}\left(z(0),t\right)$ for every t≥0. By Theorem 2, it follows that z(t) converges to an optimal input distribution as t→+∞, which is also a stationary point for the system. Moreover, if the channel admits a unique optimal input distribution, then Theorem 2 ensures that this optimal input distribution is also an asymptotically stable stationary point of the system. Besides that, the circuit computes $\sum_{k=1}^{n}z_{k}\partial_{k}I\left(z(t)\right)+1$, which equals I(z) by Proposition 1 and converges to the capacity by Theorem 2.

**Figure 2:**
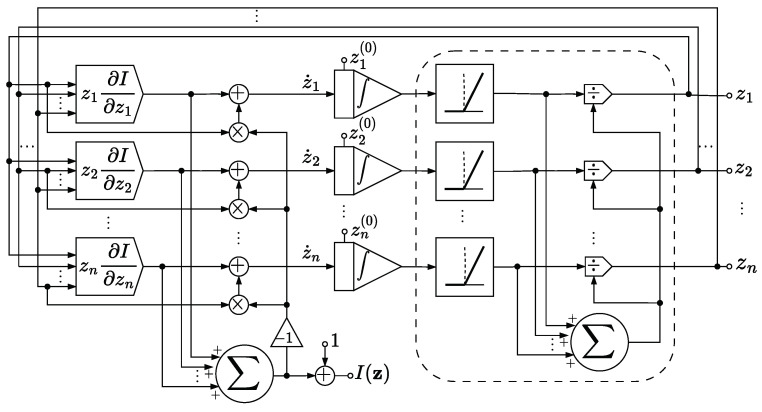
Circuit with normalizing module.

We stress that the circuit drawn in Figure 1 is supposed to work in an ideal setting, where no noise affects z. Indeed, the circuit relies on the property that $\Omega \cap \Delta_{n}$ is invariant under $\varphi_{I}$, as shown in Theorem 1. By contrast, Figure 2 shows a variation in the circuit design that may mitigate the effect of small perturbations on z thanks to an additional normalization module. The normalization module sets every negative signal received as input to zero, and then normalizes the resulting vector of non-negative signals with respect to the $\ell^{1}$-norm.

So long as z stays in $\Omega \cap \Delta_{n}$, as should theoretically happen if z(0) also is in $\Omega \cap \Delta_{n}$, then the additional normalization module has no effect on the system, and both circuits in Figure 1 and Figure 2 behave in the same way. However, in the presence of a perturbation that affects the system, then the normalization module prevents z from exiting $\Delta_{n}$. In addition to this, if the modules $z\mapsto z_{k}\partial_{k}I\left(z\right)$ are devised to implement the corresponding continuous extension described in Proposition 4, then by Peano’s existence theorem [33], the actual ODE solved by the circuit is well defined on $\Delta_{n}$, and not just on $\Omega \cap \Delta_{n}$, even though some trajectories may lead to a suboptimal input distribution, as explained in Section 9.

## 8. Some Illustrative Examples

**Figure 3:**
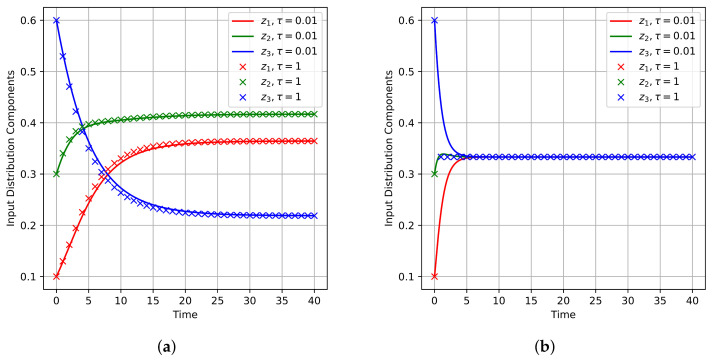
Evolution of CC-ODE and BAA orbits. (**a**) Channel with transition matrix $P^{\left(1\right)}$, systems initialized in $y={\left(0.333,0.333,0.333\right)}^{⊤}$. (**b**) Channel with transition matrix $P^{\left(2\right)}$, systems initialized in $y={\left(0.700,0.200,0.100\right)}^{⊤}$.

**Figure 4:**
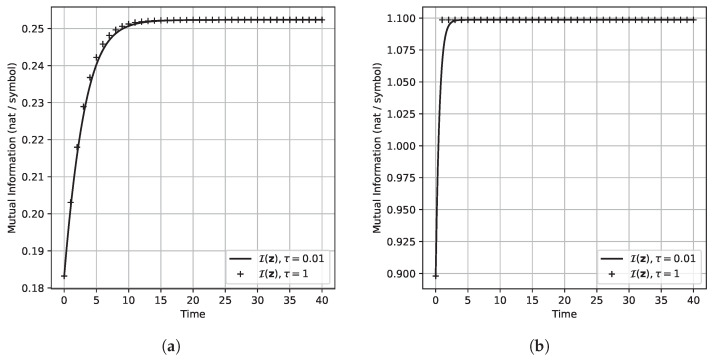
Evolution of I(z) computed along CC-ODE and BAA orbits. (**a**) Channel with transition matrix $P^{\left(1\right)}$, systems initialized in $y={\left(0.333,0.333,0.333\right)}^{⊤}$. (**b**) Channel with transition matrix $P^{\left(2\right)}$, systems initialized in $y={\left(0.700,0.200,0.100\right)}^{⊤}$.

**Figure 5:**
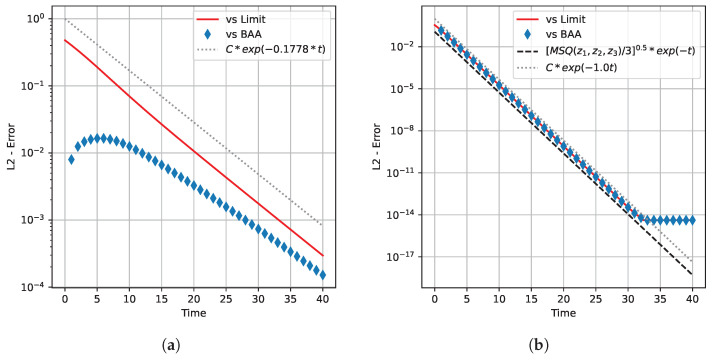
Speed of convergence of the CC-ODE: (**a**) Channel with transition matrix $P^{\left(1\right)}$, systems initialized in $y={\left(0.333,0.333,0.333\right)}^{⊤}$. (**b**) Channel with transition matrix $P^{\left(2\right)}$, systems initialized in $y={\left(0.700,0.200,0.100\right)}^{⊤}$. Diamonds are used for comparison versus the BAA and the solid red line reports the distance of z with the limit point of the dynamics. The dotted grey lines provide estimates on the minimal limiting steepness of the red line, as a consequence of Theorem 5. The dashed black line in (**b**) gives the theoretical asymptotic behavior described in Theorem 7.

To give a graphical demonstration showing the qualitative behavior of the CC-ODE flow, we simulated the CC-ODE for some specific channels. This was accomplished by applying the MWU described in (17) as an integration scheme to discretize the flow. To run the simulations, we considered two channels, and for each channel, we selected a different starting point to initialize the dynamics. For each of these configurations, we computed the (unique) optimal input distribution by running the BAA for 10,000 iterations. In particular, we considered the DMC with transition matrix

$$P^{\left(1\right)}=\left[\begin{array}{ccc} 0.70 & 0.20 & 0.10\\ 0.10 & 0.80 & 0.10\\ 0.25 & 0.25 & 0.50 \end{array}\right],$$

which admits the unique optimal input distribution $\approx {\left(0.3645,0.4169,0.2186\right)}^{⊤}$, and we considered the corresponding CC-ODE flow initialized in $y\approx {\left(0.333,0.333,0.333\right)}^{⊤}$. Similarly, we considered the noiseless and symmetric DMC with transition matrix

$$P^{\left(2\right)}=\left[\begin{array}{ccc} 1 & 0 & 0\\ 0 & 1 & 0\\ 0 & 0 & 1 \end{array}\right],$$

for which the optimal input distribution is approximately ${\left(0.333,0.333,0.333\right)}^{⊤}$, and we considered the corresponding flow initialized in ${\left(0.700,0.200,0.100\right)}^{⊤}$. To make a comparison between the CC-ODE vector flow and the BAA, we applied the MWU rule given by (17) with τ=1 and with τ=0.01, since the choice τ=1 yields exactly the Blahut–Arimoto map, whereas smaller values of τ lead to a better approximation of the CC-ODE flow. Figure 3 shows how the components of the input distribution vary as *t* runs. Note that, for both channels, the dynamics drive the system towards the optimal input distribution of the corresponding channel. Moreover, for the noiseless symmetric channel, note in Figure 3b that the BAA converges in one step. Figure 4 illustrates how the mutual information of input and output variable evolves. Also in this case, the figure displays that the CC-ODE can be used to attain the capacity, similarly to what happens for the BAA. Clearly, for the noiseless symmetric channel, the capacity is attained via the BAA with just one step—see Figure 4b. Finally, in Figure 5, we depict some graphs plotted in semilog scale to provide some estimates about the speed of convergence of the CC-ODE. For every channel considered and for every integer value of *t*, we have marked with diamonds in Figure 5 the Euclidean distance between the input distributions obtained via the CC-ODE at time *t* and the corresponding input distribution computed with the BAA. As shown in the figure, this quantity exhibits exponential decay after just a few iterations of the BAA.Moreover, we reported with a solid red line the Euclidean distance between the limit optimal input distribution and the points on the orbit produced with τ=0.01. The channels considered have a non-singular transition matrix, and their optimal input distribution does not have null entries. Consequently, Theorem 5 is applicable for both channels. As displayed in Figure 5, the solid red line decays roughly as the dotted grey line, which is the graph of a function of the form $C\exp(\lambda_{\max}t)$, with $\lambda_{\max}$ being the maximum eigenvalue discussed in Theorem 5, which equals −1 for the noiseless symmetric channel and is ≈−0.1778 for the other. For the noiseless symmetric channel, we also report in Figure 5b the theoretical asymptotic behavior described in Theorem 7 for the error decay. It is possible to see that in Figure 5b, the computed error decay deviates from the expected behavior for t≥32. However, the plot also shows that the error stabilizes when it reaches a value close to $10^{-14}$. Considering that the simulations were carried out using double precision, we interpret this outcome as a consequence of approaching the machine precision.

The interested reader may also see Ref. [29], where other integration schemes were tested in conjunction with Algorithm 1.
**Algorithm 1** Discretizing the CC-ODE to compute the capacity [29].**Input:** τ, stepsize; Niter, maximum number of iterations; ε, tolerance; y, initialization point.**Output:** $\hat{C}$, estimated channel capacity; $\hat{z}$, estimated optimal input distribution; *k*, number of integration steps performed. 1: k←0 2: z←y▹ Initializing z. 3: err←+∞ 4: **while** k<Niter∧err≥ε **do** 5:  $z_{\mathrm{new}}\leftarrow \mathrm{SolverStep}\left(z,\tau \right)$ 6:  $\mathrm{err}\leftarrow {∥z-z_{\mathrm{new}}∥}_{1}$▹ Note: ${∥x∥}_{1}=\sum_{i=1}^{n}\left|x_{i}\right|$ is the $\ell^{1}$-norm of x. 7:  $z\leftarrow z_{\mathrm{new}}$▹ Updating z. 8:  k←k+1 9: **end while**10: $\hat{z}\leftarrow z$11: $\hat{C}\leftarrow I\left(\hat{z}\right)$12: **return** $\hat{C}$, $\hat{z}$, *k*

## 9. Discussion

We report in this section some additional remarks related to the results treated so far:

- In formulating Lemma 1, we tried to require only the essential hypotheses we used in its proof. Consequently, even though we apply Lemma 1 only in Proposition 2, it is applicable also in other, more general settings.
- In the proof of Theorem 1, note that if $Y_{0}=\emptyset$, then $\Delta_{n}\subset \Omega$, and the thesis is just a mere application of classical results—see, e.g., Refs. [16,31]. By contrast, the proof deviates from a standard setting in case $Y_{0}\neq \emptyset$, which implies $\partial \Delta_{n}⊄\Omega$.
- As far as the proposed circuits are concerned, note that $z_{k}\partial_{k}I\left(z\right)$ could be produced by considering a module that outputs $\partial_{k}I\left(z\right)$ and whose output is then multiplied by $z_{k}$. However, note that $z\mapsto z_{k}\partial_{k}I\left(z\right)$ is a bounded function of the input by Proposition 4, which in general is not true for $z\mapsto \partial_{k}I\left(z\right)$.
- We also remark on the following feature of the circuit presented in Figure 1. Assume that, for whatever reason, there are *u* input symbols $x_{i_{1}}$, $x_{i_{2}}$, …, $x_{i_{u}}$ that become unavailable, in the sense that any input distribution is now constrained to assign null probability to $x_{i_{1}}$, $x_{i_{2}}$, …, $x_{i_{u}}$. Indeed, this constraint on the channel C=(X,Y,P) corresponds to replacing it with $C^{\prime }=\left(X^{\prime },Y,P^{\prime }\right)$, where $X^{\prime }=X\backslash \left\{\;x_{i_{k}}:k\in \left[u\right]\;\right\}$ and $P^{\prime }$ is obtained from P by removing the rows $i_{1}$, $i_{2}$, …, $i_{u}$. In case every symbol of the output alphabet Y can still be obtained with positive probability, then the capacity of $C^{\prime }$ may be computed without altering the circuit design displayed for C, but by simply modifying the initialization rules on z. Specifically, by Proposition 2.(d), it is sufficient to initialize $z\left(0\right)\in \Delta_{n}$ so that $z_{i}\left(0\right)=0$ if and only if $i=i_{1}$, $i_{2}$, …, $i_{u}$, and the dynamics evolves z so as to maximize I(z) under this constraint;
- Note that noise could negatively affect the capacity computation, despite the normalization module of Figure 2. Indeed, suppose that a perturbation causes z to change its support at time $t=t_{1}$, and that for $t>t_{1}$, no additional perturbations affect the dynamics. Then it is possible that z converges towards an input distribution that is still optimal, but for the wrong channel, as in the case discussed in the previous remark. To check if this has occurred, a good idea could be to consider some “perturb and restart” strategies. For instance, when the capacity of C is sought in the presence of noise, assume that a trajectory initialized in some $z\left(0\right)\in \mathrm{int}\left(\Delta_{n}\right)$ converges to some $z^{*}\in \partial \Delta_{n}$. We may then restart the dynamics in some $z\in \mathrm{int}\left(\Delta_{n}\right)$ that is obtained by perturbing slightly $z^{*}$, and then check whether I(z) converges once again to $I(z^{*})$, or if z converges to $z^{*}$ in case we know in advance that there exists a unique optimal input distribution.
- By Proposition 4 and Peano’s theorem, the ODE (7) extended by continuity admits a solution for any initialization $z\left(0\right)\in \Delta_{n}$, regardless of whether z(0) lies in Ω or not. Indeed, a solution for (7) can always be found by considering the “subchannel” of C induced by the support of z(0), similarly to the approach discussed before in Proposition 2.(d). However, in case z(0)∉Ω, note that the Picard–Lindelöf theorem cannot be applied, and we did not manage to prove that such a solution is unique. Despite this, for symmetric noiseless channels, note that the general integral (31) can be extended by continuity also for $z\left(0\right)\in \partial \Delta_{n}$, and arguing by induction it is not hard to see that, for these trivial channels, solutions for the extended ODE are unique.

## 10. Conclusions

We performed a theoretical analysis on the flow of the CC-ODE, which rules a continuous-time dynamical system that enables us to compute the capacity, as well as an optimal input distribution, of a DMC. We showed that the proposed dynamical system can be regarded as a continuous-time version of the BAA and that, under some technical conditions, the flow rate of convergence to an optimal input distribution is exponential, with constants that correspond to those arising from iterating the Blahut–Arimoto map. We described possible implementations of the CC-ODE flow in a circuit that may compute analogically the capacity of a DMC, and we discussed how the circuit may be still useful in case the channel changes due to the unavailability of some input symbols.

Possible future work stemming from this paper might be the exploration of continuous-time dynamics to maximize mutual information under some additional constraints, as in [6], or for quantum channels [41]. Moreover, an interesting aspect of Theorem 3 is that it shows that the CC-ODE flow is related to some accelerated versions of the BAA [8] that correspond to an application of (17) with f=I but where the stepsize is adjusted at every iteration. This link suggests investigating strategies to speed up analog computation by accelerating the CC-ODE flow. Lastly, a theoretical question that we have not addressed is whether solutions of the ODE obtained by extending (7) by continuity on $\Delta_{n}$ are unique, even though the hypotheses of the Picard-Lindelöf theorem are not met.

As mentioned by M. T. Chu in [42], continuous methods may help understand the corresponding discrete methods, and we hope that this paper may enrich our collective knowledge of the BAA, which is still a subject of active research.

## Data Availability

No new data were created or analyzed in this study. Data sharing is not applicable to this article.

## Appendix A. ODEs on Compact Convex Sets

Let $C\subset R^{n}$ be a closed convex set. Following Ref. [16], we recall that, for every z∈C, the *tangent cone* of *C* at z is the closed convex cone

$$TC\left(z\right)=\mathrm{cl}\left(\left\{\;\alpha (x-z)|x\in C,\;\alpha \in R_{+}\;\right\}\right)$$

where cl(·) denotes the closure in the usual Euclidean topology of $R^{n}$. We report a technical result that deals with the solutions of ODEs over *C* in case it is also compact.

**Theorem** **A1** (ODEs on compact convex sets [16] (Theorem 4.A.5.(i)))**.** *Let $C\in R^{n}$ be a compact convex set, and let $v:C\to R^{n}$ be Lipschitz continuous. Suppose that $v\left(\hat{x}\right)\in TC\left(\hat{x}\right)$ for all $\hat{x}\in C$. Then, for each ξ∈C, there exists a unique $x:[0,+\infty [\to C$ satisfying $\dot{x}=v\left(x\right)$ and x(0)=ξ.*

## Appendix B. Support Invariance and Lyapunov Function

The following proposition reports some useful properties that are known for *f* admitting a Lipschitz continuous gradient on $\Delta_{n}$. For completeness, we provide the details that extend the known proof to our more general setting.

**Proposition** **A1.**
*Let $f:R^{n}\to R$ admit a locally Lipschitz continuous gradient on some open $\Omega \subseteq R^{n}$. Let a, b∈R, with a≤b, and let the function z:[a,b]→Ω satisfy $z\left(t_{0}\right)\in \Delta_{n}$ for some $t_{0}\in \left[a,b\right]$ and*

$$\dot{z}=\left[\mathrm{diag}\left(z\right)-zz^{⊤}\right]\nabla f\left(z\right),\;a\leq t\leq b.$$

(i) *We have $z\left(t\right)\in \Delta_{n}$ for a≤t≤b;*
(ii) *The support of z(t) is invariant for a≤t≤b;*
(iii) *Either z is a constant function or f∘z is strictly increasing.*

**Proof.** (i), (ii): It is sufficient to show that the function $\sigma \left(t\right)=\sum_{i=1}^{n}z_{i}\left(t\right)$ satisfies σ(t)=1 for every a≤t≤b, and that for every i∈[n] either $z_{i}\equiv 0$ or $z_{i}\left(t\right)>0$ for every a≤t≤b. Consider first the function

$$v\left(t,s\right)=\left(1-s\right)\left[\sum_{i=1}^{n}z_{i}\left(t\right)\partial_{i}f\left(z(t)\right)\right],$$

which is defined for (t,s)∈[a,b]×R and Lipschitz continuous in *s* uniformly with respect to *t*—see, e.g., Ref. [34]. The ODE $\dot{s}=v\left(t,s\right)$ is solved by σ and admits the constant solution s≡1. Since by hypothesis $\sigma \left(t_{0}\right)=1=s\left(1\right)$, then σ(t)=s(t)=1 for every a≤t≤b by the Picard-Lindelöf theorem. Similarly, for every i∈[n], consider the function

$$u_{i}\left(t,x\right)=x\left[\partial_{i}f\left(z(t)\right)-\sum_{k=1}^{n}z_{k}\left(t\right)\partial_{k}f\left(z(t)\right)\right],$$

which is defined for (t,x)∈[a,b]×R and Lipschitz continuous in *x* uniformly with respect to *t*. The ODE $\dot{x}=u_{i}\left(t,x\right)$ is solved by $z_{i}\left(t\right)$ and admits the constant solution x≡0. Consequently, since by hypothesis $z(t_{0})\geq 0$, by the Picard-Lindelöf theorem either $x_{i}\equiv 0$ or $x_{i}\left(t\right)>0$ for every a≤t≤b. (iii): Set $\mu \left(x\right)=\sum_{i=1}^{n}x_{i}\partial_{i}f\left(x\right)$ for every x∈Ω. Note that

$$\frac{d}{dt}\left(f\circ z\right)=\sum_{i=1}^{n}\partial_{i}f\left(z\right){\dot{z}}_{i}=\sum_{i=1}^{n}z_{i}{\left[\partial_{i}f\left(z\right)-\mu \left(z\right)\right]}^{2},$$

and so

- The function f∘z has non-negative derivative, and is thereby non-decreasing.
- If for some $t_{1}$ the equality $d/\;dt\left(f\circ z\right)\left(t_{1}\right)=0$ holds, then $\partial_{i}f\left(z(t_{1})\right)-\mu \left(z(t_{1})\right)=0$ for every $i\notin \mathrm{supp}(z\left(t_{1}\right))$, and it is easy to check that in this case $\dot{z}\left(t_{1}\right)=0$—thus, $z\equiv z(t_{1})$ by the Picard-Lindelöf theorem.

□

## Appendix C. An Example

Set $z^{*}={\left(1/2,1/2,0\right)}^{⊤}$ and choose some $y\in \mathrm{int}\left(\Delta_{3}\right)$. Consider first the concave function $f_{1}\in C^{\infty }\left(R^{3}\right)$ defined by $f_{0}\left(z\right)=-{∥z-z^{*}∥}^{2}$. It is immediate to compute the Shahshahani gradient system associated with $f_{0}$:

(A1) $$\dot{z}=-2\left[\mathrm{diag}\left(z\right)-zz^{⊤}\right]\left(z-z^{*}\right).$$

Let $z_{0}:[0,+\infty [\to \Delta_{3}$ solve (A1) and suppose $z_{0}\left(0\right)=y$. Then $z_{0}\to z^{*}$ as t→+∞, and $z^{*}$ is a stationary point for (A1), as well as the global maximum of *f* over $\Delta_{3}$. Consider now the concave function $f_{1}$ defined on $R^{3}$ by $f_{1}\left(z\right)=-∥z-z^{*}∥$, and the associated Shahshahani gradient system:

(A2) $$\dot{z}=-|f_{1}\left(z\right){|}^{-1}\left[\mathrm{diag}\left(z\right)-zz^{⊤}\right]\left(z-z^{*}\right).$$

Let $z_{1}:[0,+\infty [\to \Delta_{3}$ solve (A2) and suppose $z_{1}\left(0\right)=y$. By uniqueness of solutions, $z_{1}\left(t\right)=\left(z_{0}\circ \tau \right)\left(t\right)$, with $\tau \left(t\right)=\int_{0}^{t}{\left|2f_{1}\left(z_{0}\left(r\right)\right)\right|}^{-1}dr$. Consequently, $z^{*}=\lim_{t\to +\infty }z_{1}\left(t\right)$. However, $\nabla f_{1}$ is undefined in $z^{*}$, and so (A2) is undefined in $z=z^{*}$; thus, $z^{*}$ is *not* a stationary point for (A2).

## Appendix D. Proof of Claim 1

The proof of Claim 1 here given adapts an argument used in [16] (Theorem 7.2.4), where a similar result is proved for a more stringent setting, namely, for *f* strictly convex and smooth on $\Delta_{n}$.

**Proof.** Assume that z(t) is not a constant function, since otherwise the proof is trivial. Call Λ the set of limit points of z(t) as t→+∞; then note, by compactness of *K*, that ∅≠Λ⊆K, and fix some $z^{*}\in \Lambda$. On $D_{z^{*}}=\left\{\;x\in R^{n}|\mathrm{supp}\left(x\right)⊇\mathrm{supp}\left(z^{*}\right)\;\right\}$, consider the function

(A3) $$h\left(x\right)=\sum_{i\;\in \;\mathrm{supp}(z^{*})}z_{i}^{*}\ln\frac{z_{i}^{*}}{x_{i}}.$$

Additional properties are known for h(x) if we constrain x in $D_{z^{*}}\cap \Delta_{n}$, since in this case $h\left(x\right)=D(z^{*}||x)$, where the right-hand side denotes the relative entropy between $z^{*}$ and x—see, e.g., Ref. [3]. Specifically, we are interested in the following:

- h(x)≥0, with equality only for $x=z^{*}$.
- $x\to z^{*}$ if and only if h(x)→0.

Note also that $z\left(t\right)\in D_{z^{*}}\cap \Delta_{n}$ for every *t* by Proposition A1. Therefore,

- We have h∘z≥0, and by definition of $z^{*}$,
  (A4) $$\underset{t\to +\infty }{lim inf}\left(h\circ z\right)=0;$$
- The proof is concluded if we also show that
  (A5) $$\lim_{t\to +\infty }\left(h\circ z\right)=0.$$

By Proposition A1, the function f∘z is strictly increasing; hence, by continuity of *f*,

(A6) $$f\left(z^{*}\right)=\underset{t\geq 0}{\sup}\left(f\circ z)(t\right)=\lim_{t\to +\infty }\left(f\circ z\right)\left(t\right).$$

Moreover, we can now prove that

(A7) $$\frac{d}{dt}\left(h\circ z\right)\leq 0.$$

In fact, using that $\sum_{i}z_{i}^{*}=1$, we obtain

$$\begin{array}{cc} \frac{d}{dt}\left(h\circ z\right) & =-\sum_{i\;\in \;\mathrm{supp}(z^{*})}^{n}\frac{z_{i}^{*}{\dot{z}}_{i}}{z_{i}}\\ \; & =-\sum_{i=1}^{n}z_{i}^{*}\left[\partial_{i}f\left(z\right)-\sum_{k=1}^{n}z_{k}\partial_{k}f\left(z\right)\right]\\ \; & =\sum_{k=1}^{n}z_{k}\partial_{k}f\left(z\right)-\sum_{i=1}^{n}z_{i}^{*}\partial_{i}f\left(z\right)\\ \; & =\sum_{i=1}^{n}\left(z_{i}-z_{i}^{*}\right)\partial_{i}f\left(z\right)\\ \; & =-\langle \nabla f\left(z\right),z^{*}-z\rangle \\ \; & \leq f\left(z\right)-f\left(z^{*}\right). \end{array}$$

Then (A7) follows using that

(A8) $$f\left(z^{*}\right)\leq f\left(z\right)+\langle \nabla f\left(z\right),z^{*}-z\rangle ,$$

which is a consequence of the concavity of *f* on *K*—see, e.g., Ref. [37]. By (A7), the non-negative function h∘z is non-increasing, and since we know that (A4) holds, then this yields (A5). □

## Appendix E. Proof of Claim 2

**Proof.** The Lagrangian associated with (2) is the function $L:\Delta_{n}\times R\times R^{n}\to R$ given by

$$L\left(x,\mu_{0},\mu \right)=f\left(x\right)+\mu_{0}\left(\langle 1_{n},x\rangle -1\right)+\langle \mu ,x\rangle ,$$

and the KKT conditions for $x^{*}$ amount to the following system (see, e.g., Ref. [43]):

(A9)∇xLx*,μ0,μ=∇fx*+μ01n+μ=0(A10)μixi*=0,i∈[n](A11)μ≥0.

Note that (A9) is equivalent to $\mu =-\left(\nabla f\left(x^{*}\right)+\mu_{0}1_{n}\right)$, which can be used to eliminate μ in (A10) and (A11), yielding

$$\left\{\begin{array}{cc} \left(\partial_{i}f\left(x^{*}\right)+\mu_{0}\right)x_{i}^{*}=0, & i\in \left[n\right],\\ \nabla f\left(x^{*}\right)+\mu_{0}1_{n}\leq 0, & \end{array}\right.$$

i.e.,

$$\left\{\begin{array}{cc} \partial_{i}f\left(x^{*}\right)=-\mu_{0}, & i\in \mathrm{supp}\left(x^{*}\right),\\ \partial_{i}f\left(x^{*}\right)\leq -\mu_{0}, & i\in [n]. \end{array}\right.$$

The thesis then follows by considering $\lambda =-\mu_{0}$. □

## Appendix F. Proof of Proposition 1

**Proof.** (a): Let k∈[n] and observe that

$$\begin{array}{cc} \partial_{k}I\left(z\right)\; & =c_{k}-\sum_{j=1}^{m}\partial_{k}\left(q_{j}\ln q_{j}\right)\left(z\right)\\ \; & =c_{k}-\sum_{j=1}^{m}\left\{1+\ln\left[q_{j}\left(z\right)\right]\right\}\partial_{k}q_{j}\left(z\right)\\ \; & =c_{k}-\sum_{j=1}^{m}p\left(j|k\right)-\sum_{j=1}^{m}p\left(j|k\right)\ln q_{j}\left(z\right)\\ \; & =c_{k}-1-\sum_{j=1}^{m}p\left(j|k\right)\ln q_{j}\left(z\right). \end{array}$$

(b): Apply (a) to obtain

$$\begin{array}{cc} \sum_{k=1}^{n}z_{k}\partial_{k}I\left(z\right)\; & =\sum_{k=1}^{n}z_{k}\left[c_{k}-1-\sum_{j=1}^{m}p\left(j|k\right)\ln q_{j}\right]\\ \; & =\sum_{k=1}^{n}c_{k}z_{k}-\sum_{k=1}^{n}z_{k}-\sum_{j=1}^{m}q_{j}\ln q_{j}\\ \; & =I\left(z\right)-\sum_{k=1}^{n}z_{k}. \end{array}$$

(c): By (a),

$$\partial_{i,k}^{2}I\left(z\right)=-\sum_{j=1}^{m}\frac{p(j|k)}{q_{j}\left(z\right)}\;\partial_{i}q_{j}\left(z\right)=-\sum_{j=1}^{m}\frac{p(j|i)p(j|k)}{q_{j}\left(z\right)}.$$

 □
